# Novel Organic Solvent Nanofiltration Approaches for Microbial Biosurfactants Downstream Processing

**DOI:** 10.3390/membranes13010081

**Published:** 2023-01-09

**Authors:** Miguel Figueiredo Nascimento, Petar Keković, Isabel A. C. Ribeiro, Nuno Torres Faria, Frederico Castelo Ferreira

**Affiliations:** 1 Department of Bioengineering, iBB—Institute for Bioengineering and Biosciences, Instituto Superior Técnico, Universidade de Lisboa, Av. Rovisco Pais, 1049-001 Lisboa, Portugal; 2 Associate Laboratory, i4HB—Institute for Health and Bioeconomy, Instituto Superior Técnico, Universidade de Lisboa, Av. Rovisco Pais, 1049-001 Lisboa, Portugal; 3 MIT-Portugal Program, 77 Massachusetts Avenue, Cambridge, MA 02139, USA; 4 Research Institute for Medicines (iMed.ULisboa), Faculty of Pharmacy, Universidade de Lisboa, Av. Prof. Gama Pinto, 1649-003 Lisboa, Portugal

**Keywords:** downstream processing, microbial biosurfactants, mannosylerythritol lipids, nanofiltration technology, activated carbon

## Abstract

Glycolipid biosurfactants are the most prominent group of microbial biosurfactants, comprising rhamnolipids, sophorolipids and mannosylerythritol lipids (MELs). Usually, large amounts of hydrophobic substrates (e.g., vegetable oils) are used to achieve high titers (~200 g/L) of a crude product of low purity at values limited to 50–60%, contaminated with unconsumed triacylglycerol and residual free fatty acids and monoacylglycerides. The methods reported for the removal of these contaminants use a mixture of organic solvents, compromising solvent recyclability and increasing final process costs. This study reports, for the first time, an innovative downstream method for MELs, in which 90% of the triacylglycerols are separated from the crude MEL mixture in a first stage and the other lipid derivatives (free fatty acids, mono- and diacylglycerols) are removed by organic solvent nanofiltration (OSN). Three commercially available membranes (GMT-oNF-2, PuraMEm-600 and DuramMem-500) and several homemade membranes, casted from 22, 24 or 26% (*w/v*) polybenzimidazole (PBI) solutions, were assessed for crude MELs purification by diafiltration. A final purity of 87–90% in the MELs was obtained by filtering two diavolumes of methanol or ethyl acetate solutions through a PBI 26% membrane, resulting in MELs losses of 14.7 ± 6.1% and 15.3 ± 2.2%, respectively. Higher biosurfactant purities can be archived using the PBI 26% membrane at higher DV, but at the cost of higher product losses. Namely, in MeOH, the use of 6 DV leads to losses of 32% for MELs and 18% for sophorolipids. To obtain MELs at reagent grade with purities equal or higher than 97%, a two-sequential cascade filtration approach was implemented using the commercial membrane, GMT-oNF. In such a process, MELs with 98% purity was obtained at the cost of 11.6% MELs losses. Finally, decoloration, important in some applications, was successfully assessed using activated carbon. Overall, this study reports a unique solution for microbial biosurfactants production with minimal product losses, enabling solvent recycling and potentially reducing costs.

## 1. Introduction

Microbial biosurfactants (mBS) are surfactants of microbial origin that are excreted extracellularly or incorporated as part of the cell membrane and are produced by various bacteria, yeasts and fungi [1,2]. When compared to synthetic surfactants, mostly produced from fossil fuel derivatives, mBs show higher biodegradability, are less toxic to humans and to the environment, and can be produced from renewable resources [3]. Glycolipid biosurfactants are the most prominent group of mBs due to their potency, versatility and high productivity [4]. The mBS market is evaluated at 16.5 million USD (2022) and expected to reach 24.3 million USD by 2032 [5]. Among those are the sophorolipids (SLs), produced by the yeast *Starmerella bombicola*; mannosylerythritol lipids (MELs), produced by the yeasts of the *Moesziomyces* (formerly *Pseudozyma*) genus and *Ustilago maydis* fungus; and rhamnolipids (RMs), the most explored mBS, produced by *Pseudomonas* species [1,6,7]. These glycolipids are able to reduce surface tension, even when dissolved at very low concentrations and form complex supramolecular structures. Consequentially, their potential application in a wide range of fields has been shown [8,9,10,11].

Nevertheless, the price of the cheapest mBs (34 USD/kg of SLs) is ten-fold more costly than chemical surfactants such as sodium lauryl sulphate (~1–2 USD/kg) [12]. This indicates that scalable fermentations and downstream processes are sub-optimized, and more studies are required to compete with the market of chemical surfactants. In fact, high productivity has been achieved for SLs (57.6 g·L^−1^·day^−1^) [13], but so far, most of the studies that achieved high purities (<97%) for MELs and SLs used high volumes of mixtures of solvents, not allowing solvent recyclability. Indeed, inefficient and costly downstream processing strategies are, according to some authors, the main driver of production costs for emerging bioproducts [14]. Specifically for RMs, a recent review claims that up to 80% of total production cost is allocated to downstream processing [15].

These types of glycolipids biosurfactants can be produced from a variety of substrates, including carbohydrate-based materials, lipids, as well as other compounds, such as hydrocarbons and glycerol [16]. While the highest productivity in RMs production is achieved using carbohydrate-based substrates that can be combined with hydrophobic substrates [17], MELs and SLs production uses substantial amounts of hydrophobic substrates [18,19], leading to a low purity of the final product (~30–40%), contaminated with residuals triacylglycerols (TAGs) or lipid derivatives not consumed during the fermentation process. Separating those hydrophobic contaminants from the produced MELs and SLs is challenging due to the formation of stable emulsions and other supramolecular structures (e.g., biosurfactant/water/oil systems) [20]. Therefore, the typical crude biosurfactant obtained is a product that, for most of the envisaged uses, has a low quality and applicability [21].

The processes reported for harvesting the biosurfactant fraction from the culture broth are based on organic solvent extraction, using tert-butyl methyl ether (MTBE), ethyl acetate (EtOAc) and other solvents, or sedimentation/decantation, often coupled with heating/boiling [20]. Example of chromatography column-based processes for MELs purifications are able to achieve high product purities (>97%), however, the amount of MELs recovered was limited to only 4% [22] or 50% [23], using chloroform mixtures with methanol (MeOH) or acetone as eluents, respectively. Both of these studies reported the use and disposal of large volumes of solvents, [9] particularly toxic and carcinogenic and of difficult recyclability, namely due to the formation of stable azeotropes [24]. An example of an organic solvent extraction method for MELs extractions is reported in the study by Rau et al. [25], which uses sequential liquid–liquid extraction employing 1:2 of n-hexane, 1.6:1 of MeOH, 3:1 of MTBE and 3:1 of cyclohexane (*v/v* of fermentation broth), but able to recover 8% of the MELs produced. The MTBE used in the initial extraction has the potential to be recycled by distillation, but the other solvents would require challenging separation and should be considered waste. In another study, Shen et al. [26] achieved 80% of MELs recovery employing 2.5:1 of MeOH and 3:1 n-hexane (v/v of fermentation broth) using solvent shifts (i.e., avoiding the need for solvents to be completely evaporated prior to addition of new ones). Still, solvent recyclability was hampered by the formation of complex mixtures and stable azeotropes.

Regardless of the purity obtained, all these mBSs have a distinct color (e.g., orange), which in some applications is not desired. The use of activated carbon is often adopted for product decolorization [27,28]. In this regard, Dubey et al. [29] recovered 89% of the RMs produced with a medium based on distillery wastewater, and these biosurfactants used activated carbon (AC). Several other sources [30,31] discuss the recovery of various biosurfactants by absorption using AC.

**Figure 1:**
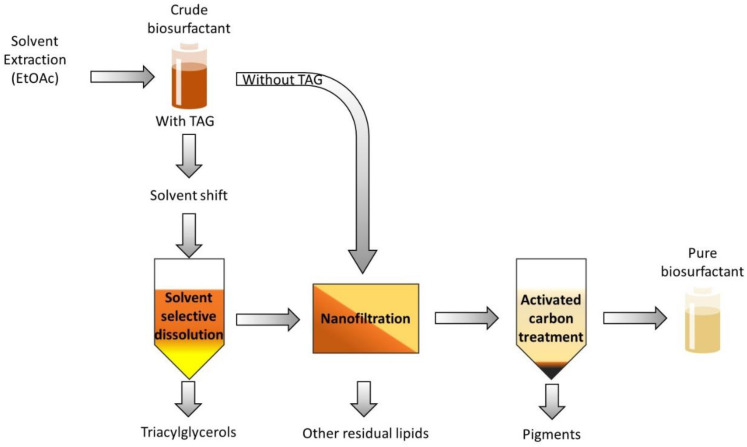
Schematic representation of the proposed downstream process for glycolipid biosurfactants produced from hydrocarbon- and lipid-based substrates.

The innovative proposed process in this study involves the use of organic solvent nanofiltration (OSN), with the goal of removing lipid derivatives (free fatty acids, mono- and diacylglycerols) smaller than MELs, RMs and SLs molecules. The separation by OSN of molecules of similar molar mass is challenging (TAGs: 870–930 Da; SL ~706 Da; RL ~650 Da; MELs ~676 Da). Therefore, prior to the OSN stage, the larger TAGs are removed by solvent-selective dissolution based on the solubility differences between MELs and TAGs in a selected solvent. Finally, AC was used to remove color impurities generated during the fermentation from the MELs, resulting in a pure product with light coloration. The downstream process is presented schematically in Figure 1. The downstream processing proposed in this work provides a unique solution for the purification of biosurfactants produced from hydrocarbon and lipid-based substrates, with emphasis on avoiding of use of solvent mixtures, allowing solvent recovery and reuse.

## 2. Materials and Methods

### 2.1. Microbial Biosurfactants

Mannosylerythritol lipids (MELs) used in this study were produced by *Moesziomyces antarcticus* PYCC 5048^T^ (CBS 5955) and *M. aphidis* PYCC 5535^T^, provided by the Portuguese Yeast Culture Collection (PYCC), CREM, FCT/UNL, Caparica, Portugal, following previously established fermentation medium and conditions [19]. Yeasts were cultivated in a 2-L bioreactor (New Brunswick^TM^ Bioflo^R^/CelliGen^R^ 115), using 1.5 L of working volume, with a medium composition described elsewhere [32]. The temperature was set up at 27 °C and the pH was not controlled. The dissolved oxygen (DO) was set up for 15%, varying the agitation and the air-flow rate in the ranges from 150–800 rpm and 0.5–1.5 vvm, respectively. After 12 days of cultivation, the broth was extracted three times with EtOAc in equal volumes to the fermentation broth and the solvent was evaporated and recovered using a rotavapor. The composition of the crude MELs vary widely with the feed strategy used. Typical examples are described on Table 1. Here we consider three case studies that include: (A) the standard co-substrate feeding strategy optimized to render a TAG free product [33]; (B) a non-optimized co-substrate feeding strategy [34], yielding crude MEL with high content on FFA and MG, but negligible TAG; and (C) the feeding strategy based on one single addition of vegetable oil at day zero, yielding a crude MEL with TAG [35].

Note that such compositions are presented for indicative purposes, as the exact values are batch-dependent. Therefore, when crude MELs of different compositions are used, the initial MELs purity before the separation step is indicated in the respective data set. Sophorolipids (SLs) were provided by Holiferm, UK, and were also used to assess the separation strategies proposed in the current study.

### 2.2. Lipids Hydrolysis as MELs Extract Contaminants

Commercial immobilized CAL-B (Novozym^®^ 435, Novozymes, Copenhagen, Denmark) enzymes, diluted in MilliQ water, were used for the hydrolysis of lipids and their derivatives. This mixture was kept at 50 °C and mixed with a magnetic stirrer at 400 rpm for up to 24 h, when the organic phase was collected. This mixture of lipids and lipid derivatives was characterized, and mixed with biosurfactants in different ratios, to simulate the extracts collected from fermentations with residual lipid impurities.

### 2.3. Analysis of Fatty Acids, Mono-, Di- and Triacylglyceride Concentrations

Fatty acids, mono-, di- and triacylglycerides contents were determined by high-performance liquid chromatography (HPLC), following a method developed by Badenes et al. [36]. Samples of supernatant (200 μL) were mixed with 1 μL of acetic acid 58.5 mM and 799 μL of n-hexane, and centrifuged at 10,000 rpm for 2 min. The organic phase was recovered and used for HPLC analysis using a Chromolith Performance RP-18 endcapped (100 mm × 4.6 mm × 2 μm) column with a UV detector at 205 nm. The injection volume was 20 μL. Three mobile phases, at 1 mL/min, were employed: phase A = acetonitrile 100%, phase B = water 100% and phase C = n-hexane/2-propanol (4:5, *v/v*).

### 2.4. Analysis of MELs, SLs and Residual Lipid Concentrations

MELs and residual lipids from the feed, permeate and retentate solutions were quantified after solvent evaporation, following the methods previously described, through GC analysis of methyl esters generated by methanolysis of the solutes [37]. SLs were measured by MS-HPLC as described elsewhere [38].

### 2.5. Thin Layer Chromatography (TLC)

Samples of crude MELs, MELs, lipids and other solutions were eluted using various solvents, such as isopropanol (IPA), chloroform, tert-butyl methyl ether (MTBE), methanol (MeOH), ethyl acetate (EtOAc), dichloromethane (DCM), n-hexane, water, ethanol (EtOH) and acetone, in a closed TLC development chamber. The standard solvent mixture used for the separation of different MELs homologues including the use of a solvent system of chloroform/MeOH/water (6.5:1.5:0.2) as an eluent. Precoated aluminum TLC sheets with a silica gel 60 coating were used (Macherey-Nagel Alugram Xtra SIL G/UV_254_) with 0.5–1.0 mg (dw) of the analyzed sample being placed for each lane on the TLC. To visualize the compounds, a developing solution of α-naphthol in sulfuric acid (1.5 g of naphthol, 51 mL of ethanol, 4 mL of water and 6.5 mL of sulfuric acid) was sprayed, and the plate was heated over 100 °C for 1 min. Further assessment of the separation of MELs from TAGs, based on the difference in their solubility in a selected solvent, is described in the Results and Discussion sections.

### 2.6. Membrane Preparation

A homemade polybenzimidazole (PBI) organic solvent membrane (OSN) was manufactured by the phase inversion technique. Celazole^®^ S26 solution (26 wt% PBI, 1.5 wt% LiCl in N,N-dimethylacetamide (DMAc), PBI Performance Products Inc., North Charleston, SC, USA) was diluted with DMAc (Panreac, Barcelona, Spain) to 22, 24 and 26 wt% PBI concentration. The solution was mechanically stirred at 60 rpm overnight to obtain a homogeneous dope solution, and an additional 24 h unstirred for the removal of air bubbles. The resulting solution was first manually casted using a homemade casting knife, height of 250 µm, on the top of a non-woven Polyolefin Novatexx 2471 (Freudenberg Filtration Technologies, Weinheim, Germany), selected to provide additional mechanic support of the PBI membranes, without significant addition of mass transfer resistance. The film obtained was then immersed in a distilled water precipitation bath (1 h, three times), and then in an IPA (Carlo Erba, Barcelona, Spain) bath (1 h, three times) for water removal. The obtained membranes were kept on IPA until further use. All the processes were performed at room temperature and 40% humidity.

The cross-linking of the above-described OSN membranes was also considered following a post-manufacture treatment of the membrane. A cross-linking solution was prepared by mixing 3% of α,α,-Dibromo-p-xylene (Sigma Aldrich, St. Louis, MO, USA) with acetonitrile. The membranes prepared and stored in IPA were immersed and agitated in the cross-linking solution for 24 h at 80 °C under reflux. After 24 h, the reaction was stopped and the membrane was washed three times with IPA, to remove residues of the cross-linker solution and solvent. All membranes were stored in IPA. The cross-linked PBI membranes were labeled as PBI-X.

### 2.7. Membrane Characterization and Microbial Biosurfactants Purification by OSN

The commercially available OSN membranes assessed in this work were (i) the GMT-oNF-2 (Borsing GmbH, Berlin, Germany), DuraMem-500 (Evonik, Essen, Germany) and PuraMem-600 (Evonik), with molecular cut-off (MWCO) of about 600, 500 and 600 Da, respectively; and (ii) the homemade PBI and PBI-X OSN membranes, made as previously described.

The filtrations were performed on a dead-end Sterlitech HP 4750 Stirred Cell, fitted with a circular OSN membrane with an area of 14.6 cm^2^. Duplicates were performed using different membrane samples, which were preconditioned by filtering pure solvent (~400 mL) until a constant solvent flux was obtained. A pressure of 15 bar was applied using pressurized nitrogen gas, and all experiments were performed under magnetic stirring of 300 rpm at room temperature. Concentrations of MELs and residual lipids on OSN studies were assessed by GC as previously described. The experimental strategy followed used crude MELs, with typical compositions based on the one described in Table 1. Crude MELs were dissolved in either EtOAc or MeOH at 50 g/L, and 50 mL of the solutions obtained were used as feed solution for MELs rejection estimation and MELs purification by diafiltration.

The solvent flux (ϕ) was estimated by Equation (1), based on membrane area *(*Am), filtration time (t) and permeate volume (V_P_). The membrane rejection (R) was estimated by Equation (2), considering solute concentrations in the feed (C_F_) and permeate (C_P_), after permeation of 50% of the feed volume in concentration mode.

(1) $$\phi =\frac{V_{P}}{A_{m}\times \;t}$$

(2) $$R=1-\frac{C_{P}}{C_{F}}$$

MELs purifications were assessed in diafiltration mode, allowing the retention of the product, as smaller lipidic molecules were pushed through the OSN membrane. An HPLC pump Series I, Scientific Systems Inc., was used to add fresh solvent (EtOAc or MeOH) as required to keep the retentate nanofiltration cell volume constant at a value of 50 mL, compensating for the volume leaving the system through the permeate. Diavolume (DV) is defined as the volume of fresh solvent added by the retentate constant volume (i.e., the initial volume of crude MELs solution submitted to OSN). Samples were collected to measure the solute concentrations on the retentate (C_R_) and permeate (C_P_), after the addition of 2, 4 and 6 DV of solvent, i.e., after permeation of 100, 200 and 300 mL of permeate. MEL losses or contaminants removal can be calculated by Equation (3), according with membrane rejection to the solute and DV used.

(3) $$\frac{C_{R}}{C_{F}}=e^{-\mathrm{DV}\left(1-R\right)}$$

### 2.8. Coloration Analysis and Product Purification Using Activated

Absorbance at 450–650 nm wavelength spectres were determined in freshly made samples, autoclaved samples, and autoclaved samples treated with 1% activated carbon. Autoclavation was carried out in an autoclave (AJC, Uniclave 88) for 20 min at 121 °C and 1 bar, following manufacturer instructions. For activated carbon (AC) treatment, the autoclaved samples were added to falcon tubes with 1% (*w/w*) AC, vigorously mixed using a vortex mixer for 30 s, and then centrifuged for 8 min at 10,000 rpm to precipitate AC. The supernatant was separated, 200 μL samples of it were collected and measured in a spectrophotometer (Hitachi U-2000) for absorbance between 450 and 650 nm. To the absorbance value measured for each sample, we subtracted the value obtained by a blank measurement containing the solvent (water, EtOAc or MeOH).

### 2.9. Statistical Analysis

Statistics were performed by the analysis of variance (two-way ANOVA), and *p*-values of the differences between groups were corrected for simultaneous hypothesis testing according to Tukey’s method. The level of significance was set at *p* < 0.05.

## 3. Results and Discussion

A novel multi-step downstream process, a first stage combining TAG selective removal from glycolipids and a second stage using OSN for molecular separation of the glycolipid from smaller lipidic impurities, is proposed here for the purification of glycolipids biosurfactants produced from lipid-based substrates. Finally, a stage of product decoloration using activated carbon was also assessed.

OSN is assessed here with the aim of removing free fatty acids and monoacylglycerols of smaller molecule size. Those are predominant contaminants on longer fermentations, or the ones optimized to avoid overfeeding of vegetable oils used as substrates (e.g., Table 1, strategy A), which have a low content on TGA. For such case studies, downstream route can employ ethyl acetate (EtOAc) as the same solvent for crude MELs extraction from the fermentation broth and in the OSN.

Noteworthy, TAGs have similar or larger molecular weights than glycolipids biosurfactants. Therefore, for fermentations with large contaminations on TAG (e.g., Table 1, strategy B), a stage prior to OSN to remove these large molecules are advisable. Such separation has been attempted by gravimetry for SLs [12], but such a process is not efficient for MELs. In the current study, TAG removal from MELs by the solvent-selective dissolution is studied. Again, ideally, we will seek to use the same solvent on TAG removal and in OSN stages.

Purification of crude MELs was selected as the case study and proof of concept for the novel technological approach suggested here, but the OSN stage was then also assessed with SLs.

### 3.1. Selection of Solvent for MELs Separation from TAGs

#### 3.1.1. Solvent Interaction with MELs and Lipids

The use of large quantities of vegetable oil or other hydrophobic carbon sources to improve MEL titers often leads to the contamination of the final product with significant amounts of unconsumed TAG and/or other residual lipids (Table 1). The first study assessed the interaction of organic solvents with MELs and TAG, aiming at their efficient separation by thin-layer chromatography (TLC). TAGs mainly comprise soybean oil (SBO), and thus, the TLC reference values (Rf) for SBO and with ~85% purity (Table 1, feeding strategy A, without TAG present) in different solvents were measured. The results for the tested eluents are represented in Table 2. TLCs can be found in Figure S1.

The higher the differences between Rfs values (ΔRf), the higher the differences in solvents’ affinity to MELs and TAGs, and thus, the higher potential of using such solvents in these compounds’ separation. Dichloromethane (DCM) and chloroform present the higher ΔRf, but given their poor environmental performance, the extracting solvent was selected from the best performing non-halogenated ones, i.e., ethanol (EtOH), methanol (MeOH) and tert-butyl methyl ether (MTBE). Ideally, the selected solvent should be possible to be produced from renewable resources in a sustainable manner, and, importantly to be able to separate MELs and TAGs by dissolving and eluting one of the compounds at high concentrations while not solvating the other one and thus promoting its precipitation as a separating layer. This would allow implementing a “washing” step, to remove TAGs from the crude MELs.

#### 3.1.2. The Role of Methanol in MELs Separation from TAGs

MeOH was selected as the best candidate for separation of TAG and MELs, in spite of other solvents have presented a higher ΔRf, considering chloroform or DCM have been exclude due to their chlorohydrocarbons nature and volatility. MTBE and EtOH were not efficient on discriminating between TAG and MELs on their elution/precipitation [39,40]. On the other hand, MeOH dissolves MELs and free fatty acids completely (Figure S2), while almost no TAG can be dissolved in it, enabling the separation. Indeed, the solubility of SBO in MeOH was negligible (<1 g/L), however, separation efficiency drops with higher concentrations of MELs, as the biosurfactant and the oil probably form macromolecular structures, stabilizing the oil in the methanol solution.

The removal of TAGs from MELs, exploring the different compound solubility in MeOH, was then studied. Crude MELs (feeding strategy B) and SBO, accounting for a total mass of 8.3 g, were vigorously mixed in 20 mL of MeOH. The mixture obtained represents a case study where MEL is highly contaminated with TAGs—indeed the solute mass fractions of MELs and TAG were similar (~39% each), and the remaining solute fraction of 22% accounts for ~7% free fatty acids (FFA), ~14% monacylglycerides (MAG), and ~1% diacylglycerides (DAG). The mixture was then submitted to a mild centrifugation (2 min at 4000 rpm) to speed up phase separation. The top solution, enriched in MELs, was separated and kept as a purified fraction, while TAGs were predominantly left in the bottom layer, a precipitate. Additional washings can be performed to increase triacylglycerol removal with additional MELs losses.

Importantly, a single MeOH washing step allowed, by selective dissolution, to remove about 94% of TAG from the crude MELs, with a product loss of 12.3%. (Table 3, step 1). This separation was performed at concentrations of ~34 wt% total solute, i.e., ~21 wt% MELs, in MeOH. However, such separation was not optimized and higher MELs purities could be achieved using higher solute concentrations obtained using lower volumes of MeOH. In such conditions, higher amounts of TAGs will precipitate, but eventually, some additional MELs will be lost to the bottom phase.

On the other hand, MELs losses could be mitigated by using higher volumes of solvent or applying consecutive steps of MeOH washing to further retrieve the lost MELs from the bottom layer. Such a strategy was experimentally applied by redissolving in MeOH, the bottom layer obtained in the previous step. The phases were separated as previously described. This was repeated two times. The results of the analysis of the phases obtained in this three-step separation process are presented in Figure S3. The percentages of components in each fraction are shown, while the totals indicate the total dry mass of the sample. The values of the cumulative losses in MELs (Equation (4)), cumulative removal contaminants (Equation (5)), and the composition of the combined top phases are presented in Table 3.

(4) $$\mathrm{Cumulative}\;\mathrm{MEL}\;\mathrm{Losses}\;\left(\%\right)=\left(1-\frac{\sum_{i=0}^{n}\mathrm{MEL}{\left(g\right)}_{\mathrm{Top}\;\mathrm{phase},\;i}}{\mathrm{MEL}{\left(g\right)}_{\mathrm{initial}\;\mathrm{sample}}}\right)\times 100$$

(5) $$\mathrm{Cumulative}\;\mathrm{contaminant}\;\mathrm{removal}\;\left(\%\right)=\frac{\sum_{i=0}^{n}\mathrm{TAG}{\left(g\right)}_{\mathrm{Top}\;\mathrm{phase},\;i}}{\mathrm{TAG}{\left(g\right)}_{\mathrm{initial}\;\mathrm{sample}}}\times 100$$

The results obtained point out that it is possible to reduce MEL losses to values of 3.55%, maintaining MELs purity at values above 90%. Importantly, FFA and MAG are preferentially dissolved together with MELs on the top MeOH solution. In the case of the experiment performed here, after removal of the TAGs, 30% of the total weight of the product is comprised of FFA and MAG (Table 3). Therefore, there is a need to design a second separation stage to remove these contaminants.

### 3.2. Nanofiltration as a New Downstream Route for MELs Purification

The technological challenges for organic solvent nanofiltration (OSN) and aqueous stream nanofiltrations are slightly different. For aqueous systems, one of the main issues is fouling at the membrane surface, due to the combined action of ions (e.g., Ca^2+^) and of organic matter (e.g., driven from cells debris, proteins, etc., that stay in the aqueous phase). On the other hand, for OSN the use of membranes stable on the solvents used, i.e., without swelling and able to maintain their performance, is of utmost importance. In the last two decades, the development of OSN membranes have been extensively evolved and have been assessed for the separation and purification of many compounds [41]. In our research group, a special focus has been given to the separation and purification of API (active pharmaceutical ingredients), especially using PBI membranes [42]. Therefore, in the current study, we decided to compare the efficiency of commercially available OSN membranes against homemade PBI membranes.

In this study, the OSN membrane is used to retain MELs (MW ranging from 580–650 Da), while smaller residual lipids, e.g., FA and MAG (280–350 Da) are pushed into the permeate. The diafiltration should be carried out in a solvent which, to facilitate its recycling, should be the same that is used in the previous step of the downstream process. Such solvents are either (i) MeOH, used for removal of TAGs (e.g., Table 1, feeding strategy A) or (ii) EtOAc used for MELs extraction from the fermentation broth (e.g., Table 1, feeding strategy B). This latter case is particularly relevant for fermentations optimized for complete TAG metabolization with residual smaller lipid derivatives (e.g., FFA and TAM) left unconsumed.

In summary, the OSN membranes selected should be compatible with MeOH or EtOAc and have MWCO high enough to retain MELs. Therefore, considering the existing knowledge on OSN membranes, the membranes selected for this study were the three commercially available membranes, GMT-oNF-2 (based on polydimethylsiloxane, MWCO 600 Da), PuraMem 600 (polyimide, MWCO 600 Da) and the DuraMem-500 (cross-linked polyimide, MWCO of 500 Da) and homemade PBI membranes casted from solutions with 22, 24 and 26% PBI solutions, as prepared or cross-linked. These membranes have been previously characterized by Razali et al. [43] for their permeability in different organic solvents, such as EtOAc, MeOH, toluene and rejection of specific compounds with different molecular sizes.

In the current study, the membranes were assessed by estimating solvent fluxes, for EtOAc and MeOH, through the membrane and the membrane rejections to MELs and lipids present on the crude MELs. Those values were then imputed for theoretical estimations of MELs losses and diavolumes needed to obtain MEL at a reagent grade of 97%, starting from a crude MEL with a purity of 85%. Finally, the more promising OSN membranes were experimentally assayed for crude MELs purification, removing the smaller lipidic derivatives by diafiltration in MeOH or EtOAc. A sequential cascade OSN system was also assessed, aiming at higher MELs purities, with the permeate of the first diafiltration fed to a second diafiltration to improve overall MEL recovery yields.

#### 3.2.1. Membrane Screening

**Figure 2:**
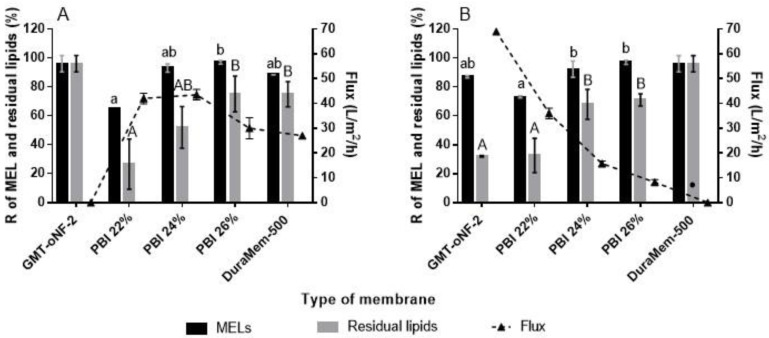
The rejection coefficient for MELs and residual lipids, and the solvent flux for each OSN membrane: PBI 22, 24 and 26%; GMT-oNF-2 (Borsig) and DuraMem-500 (Evonik) using MeOH (**A**) and EtOAc (**B**). Dots refer to filtrations that did not occur, since no solution has permeated. Different lowercase and uppercase letters in each colunm represent significant variations in MELs and residual lipids rejections, respectively, for different membranes and the same solvent. Results for membranes highlighted in different letters have a *p*-value lower than 0.05.

The solvent fluxes through the membranes were estimated by Equation (2) (Figure 2 and Table S1). In the case of commercial membranes, it was interesting to observe that solvent fluxes of MeOH (5.1 of polarity index) and EtOAc (4.1 of polarity index) were negligible (lower than 0.3 L·m^−2^·h^−1^) for the GMT-oNF-2 (based on polydimethylsiloxane) and the DuraMem-500 (cross-linked polyimide) membranes, respectively. This is explained by the interaction of the solvent with the membrane, as discussed by Razali et al. [43], showing that the solvent–polymer interaction is crucial for the success of the filtration. Thus low permeability is observed for more polar solvents (i.e., MeOH) through hydrophobic membranes (GMT-oNF-2) or more apolar solvents (i.e., EtOAc) through hydrophilic membranes (e.g., DuraMem-500). The most commonly used process for cross-linking polyimide makes this polymer more hydrophilic. Therefore, a negligible EtOAc flux was observed for DuraMem-500 (cross-linked polyimide membrane), but a 25.5 L·m^−2^·h^−1^ EtOAc flux was measured through PuraMem-600 (non-crossed linked polyimide membrane) under an applied pressure of 15 bar. The EtOAc fluxes of the homemade PBI membranes decrease, from 36 to 9 L·m^−2^·h^−1^, with increasing concentration of PBI casted solution from 22% to 26%. The observed MeOH fluxes are higher and more independent of PBI concentrations, ranging between 30.0 to 43.5 L·m^−2^·h^−1^, as PBI is a hydrophilic polymer, and so the interaction with MeOH is higher.

Retention of the larger product to purify is typically highlighted as the main decision criteria for the selection of the membrane. However, as previously illustrated mathematically by Ferreira et al. [44], the high rejection of impurities to be pushed through the membrane is also a main obstacle to efficient separations. While MWCO is a valid metric concerning the retention of larger molecules, the permeation of the smaller ones depends on the shape of the retention curve, which is often not sharper enough for a successful separation. The rejection coefficients (R) were calculated (Equation (1)) for MELs and residual lipids (Figure 2 and Table S1). A high-performance membrane is one that has a high rejection coefficient for MEL and a low rejection coefficient for residual lipids, allowing a better separation between the products presented in crude extracted MEL.

Concerning the use of commercially available membranes, the rejection of DuraMem-500 for residual lipids in MeOH is 77%, making this separation very challenging as it is difficult to push these solutes through the membrane. On the other hand, GMT-oNF-2 has a rejection of residual lipids of 32% in EtOAc, and thus it has the potential to separate them from MELs. Still, the forecast product losses may be significant as this membrane presents a rejection to MEL of only 87.1%.

The PBI 22% membrane, with residual lipids rejection coefficients of 26.6% in MeOH and 32.6% in EtOAc has the potential to effectively remove these compounds’ residual lipids from MELs. However, again the rejection coefficients for MELs are low, at values of around 67.7% and 78.4%, respectively, for MeOH and EtOAc.

In order to increase MEL retention, a decision was made to increase the percentage of PBI in the casting solution and to assess the cross-linking of the membrane after its manufacture. The intended effect was observed, with PBI 24 and 26% membranes revealing higher MELs rejection (>90%), with no significant differences between both solvents, EtOAc or MeOH (Figure 2 and Table S1). However, the rejection coefficients for residual lipids also increased significantly for PBI 24% and PBI 26%, reaching values of 68% and 71% in EtOAc and 52% and 75% in MeOH, respectively.

Cross-linking PBI membranes typically leads to decreased membrane MWCO and increased robustness, as shown by Valtcheva et al. [45]. As expected, also in our study, the cross-link of PBI membranes lead, in the most cases, to a decrease in solvent fluxes and an increase in MELs rejections. However, PBI membrane cross-linking also leads to a significant increase in residual lipids rejection, an increase which was particularly stringent for PBI 22%. Such an effect is not beneficial for the intended separation; therefore, the use of PBI cross-linked membranes was not further considered in this study.

Importantly, note that average rejection coefficients are estimated with significant associated standard deviations (Table S1), especially for MELs rejection. Such variation results from the potential variations in pore size distribution on the membrane active layers due to slightly different environmental conditions in the phase inversion process, leading to different rejections from batch to batch. Moreover, according to fermentation variability, different MELs congeners and residual lipids, which can be presented as free fatty acids or acylglycerides, will be fed to the filtration. While similar rejection was measured for MELs, regardless of their fatty acid chain length, the rejection of residual lipids presented higher variabilities (Figure S4).

#### 3.2.2. Diavolumes Strategy for MELs Purification

In this study, MELs purification will be assessed following a diavolume (DV) strategy, aimed at retaining and purifing MELs, while pushing the smaller lipids molecules through the OSN membrane. In this regard, the minimum theoretical DV and the MELs losses associated for each membrane were calculated to achieve (at least) 97% of MEL purity by considering the rejection coefficients values obtained for MELs and for residual lipids (Figure 2) as well as a crude MEL with 85% purity, broadly corresponding to the case studies identified on Table 1 as “feeding strategy A” in EtOAc or “feeding strategy C”, after removal of TAGs by MeOH selective dissolution.

**Figure 3:**
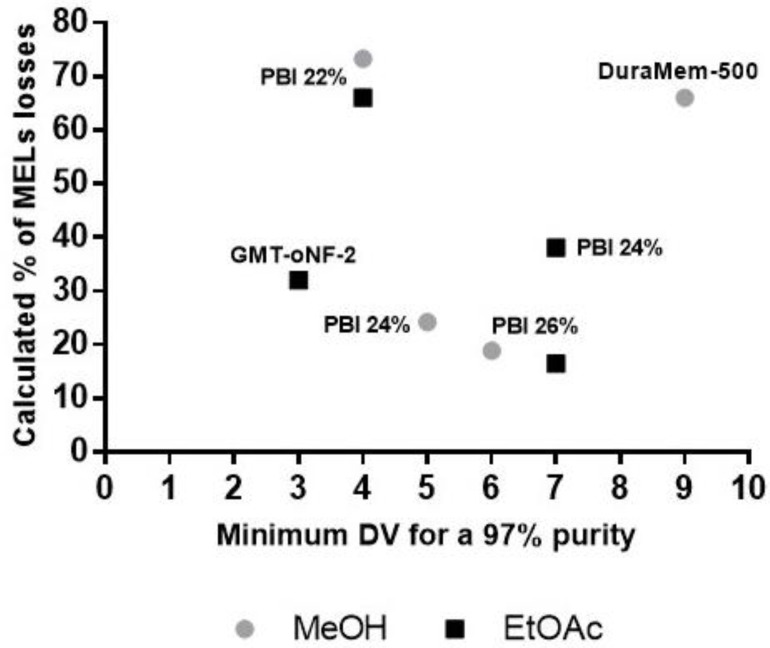
Theoretical minimum DV vs. calculated MELs losses for each OSN membrane: PBI 22, 24 and 26%; GMT-oNF-2 and DuraMem-500, for MeOH (grey circles) and EtOAc (black squares) as organic solvents, to achieve at least 97% of MELs purity. All these results were calculated using Equation (2) and the values shown in Figure 2 and Table S1.

In Figure 3, it is possible to observe that filtrations presenting lower theoretical MELs losses (ranging from 13–16% losses) were the ones using PBI 26% membrane, which theoretically requires 6 to 7 DV to reach the 97% MEL purity. This membrane was selected to be experimentally assessed under diafiltration mode.

The poorest performances were estimated for filtrations using PBI 22% and GMT-oNF-2 membranes, with MELs losses ranging from 65–80%. Nevertheless, since these OSN membranes (PBI 22% and GMT-oNF-2) had a higher MWCO than PBI 26%, lipids permeation was less challenging and only 4 DV were necessary to achieve 97% purity. The use of such membranes could be interesting for a cascade membrane system, where MELs, lost to the permeate on the first step, would be recovered in the second step. Therefore, their performance, under diafiltration mode, was also experimentally assessed.

**Figure 4:**
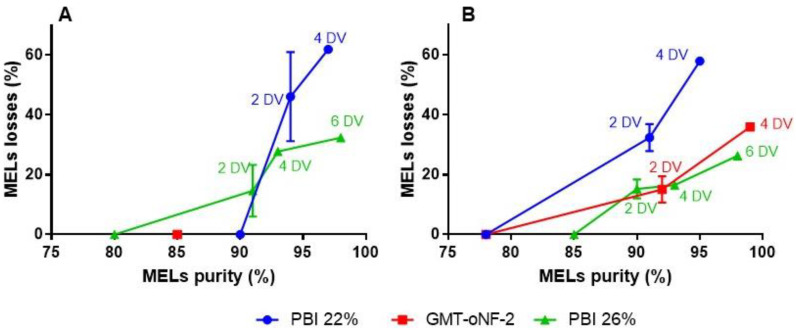
MELs purification through DV technology for different OSN membranes (PBI 22 (blue line with circles) and 26% (green line with triangles), and GMT-oNF-2 (red line with squares), using MeOH (**A**) or EtOAc (**B**) as organic solvents, for different DV (0, 2, 4, 6). All filtrations were performed by maintaining a volume of 50 mL and 15 bar of pressure.

The experimental results for MELs purity and losses obtained for each DV using for PBI 22, 26% and GMT-oNF2 membranes, are shown in Figure 4.

When using PBI 26%, the best-performing membrane, there were no differences in the final MEL purity attained with MeOH and EtOAc, reaching more than 97% MELs purity and losses of 26.3% and 32.4% for EtOAc and MeOH, respectively, after 6 DV. These values are still high compared to the best value reported, 10% MELs losses, which used several solvent extractions to reach similar purities [26].

Remarkably, after 2 DV of EtOAc, a diafiltration using GMT-oNF-2 or PBI 26% resulted in a purity higher than 90%, with losses of 15.08 ± 3.10% and 15.3 ± 2.2%, respectively. Again, a similar performance, corresponding to MELs losses of 14.7 ± 6.1%, could be achieved by diafiltration of 2 DV of MeOH through the PBI 26%.

The strategy presented here can be an interesting purification platform for other biosurfactants, particularly glycolipids, that face similar challenges in the separation of lipidic fractions from the glycolipids. As a proof of concept for other microbial biosurfactants, a solution of 50 g/L of crude SLs with 87% of purity was diafiltrated using PBI 26% with MeOH, as SLs do not dissolve well in EtOAc. After 6 DV, it is possible to purify SLs (Figure S5), and with losses of around 18%. A value lower than the one obtained for MELs losses after a 6 DV diafiltration also performed in MeOH and using the PBI 26% membrane, which is expected considering the larger size of SLs (MW 650–685).

Envisaging membrane cascade systems, when using GMT-oNF-2 or PBI 22% membrane, after diafiltrating 4 DV of EtOAc, 36.0 or 58.0% of the MELs will be in the permeate, while the MELs on the retentate will have purities of 99% or 95%, respectively. For MeOH-based diafiltrations, the PBI 22% with 2 DV could be used to obtain MELs in the retentate with 98% purity, while 62% of MELs will be in the permeate. MELs losses theoretically and experimentally estimated are quite similar for PBI 22% and GMT-oNF-2 (coefficients of variation of 3–12%) but more divergent for the PBI 26% (coefficients of variation between 20–26%). A possible explanation is related to the fact that rejection coefficients were obtained in concentration mode with a decrease of the retentate volume to half of its initial value, while in the diafiltration, the retentate volume was kept constant.

#### 3.2.3. Cascade System for Reduction of MELs Losses

As discussed, the use of the membranes assessed in this study were far from ideal for the separation intended. The membranes that provide high retentions to MELs (above 97%), also present high rejections for the lipids (above 70%), implying the need of using high DV, which over the extensive diafiltration leads to cumulative substantial losses in MELs to the permeate, as illustrated on the previous section for PBI 26%. A possible strategy to obtain high product purity with low losses would be the development of new membranes with better performance, i.e., lower MWCO able of higher retentions of MELs and sharper rejection curves with lower rejections for lipids.

**Figure 5:**
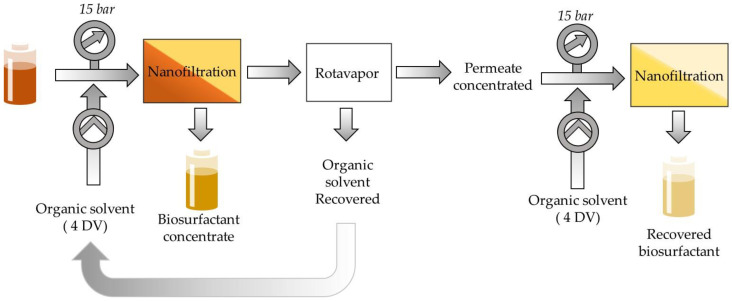
Overview of the cascade system studied, which included a first diafiltration, the recovery and concentration of the filtrate, and a second diafiltration using the filtrate as feed solution. Both diafiltrations used 4 DV.

However, here we follow the non-obvious approach, and we suggest the use OSN membranes, such as GMT-oNF-2 and PBI 22%, that have lower MELs rejections (67.7 to 87.1%), but also with lower rejections to the lipids (26.6–32.6%). In other words, to facilitate the removal of lipids, we indulged in a separation with high MELs losses to the permeate. Such a strategy, if implemented on a single step, will lead to non-acceptable losses of MELs. Still, a two-step filtration cascade OSN membrane system, as described by Kim et al. [46], can be implemented, where the permeate of the first filtration is fed into a second filtration to recover the MELs, while the lipids is pushed into the permeate. Such a system was assessed using the same membrane for the consecutive DV filtrations, as represented in Figure 5, which illustrates the two diafiltrations with an intermediate step of solvent evaporation to concentrate the permeate (with MELs and residual lipids). The solvent distilled from both permeates can be recycled as fed solvent into the diafiltration. The DV was selected to be the lowest number to achieve >95% of MELs purity, regardless the MELs losses, which for the selected membranes correspond to 4 DV.

**Figure 6:**
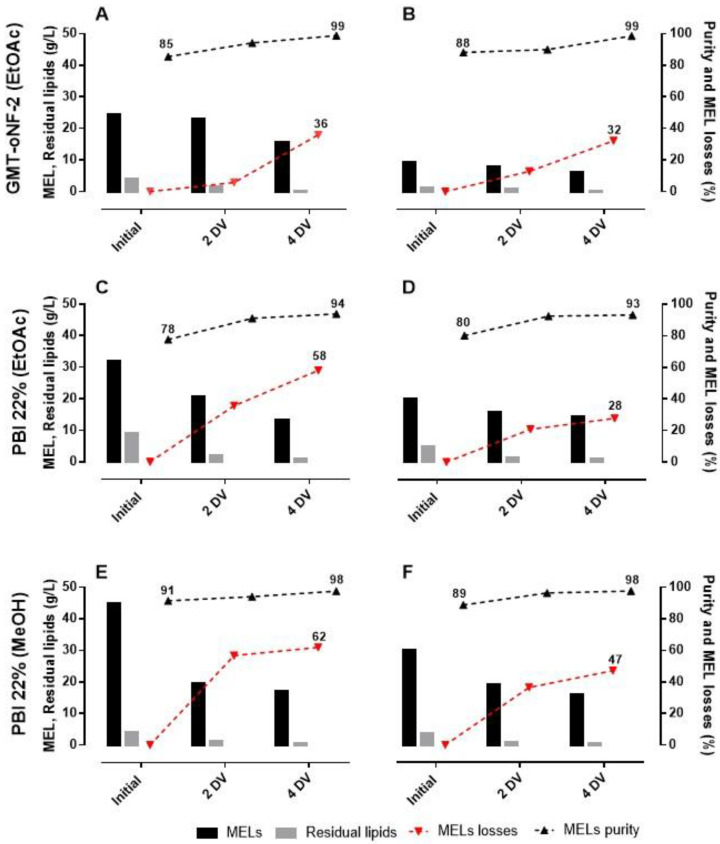
Cascade system using OSN membranes: GMT-oNF-2 (**C**–**F**) and PBI 22% using EtOAc (**A**–**D**) and MeOH (**E**,**F**) as organic solvents. After the first filtration (**A**,**C**,**F**) the permeate was concentrated and passed again through the same membrane (**B**,**D**,**E**). Each graphic represents the concentration of MELs in the retentate (black bar), residual lipids (grey bar), MELs losses (red line with inverted triangles) and purity (black line with triangles). For all filtrations used, the volume was kept at 50 mL, and 15 bar of pressure was applied.

Figure 6 shows MELs concentration in the retentate for each DV, as well the purity and losses associated for each cascade system. While the first diafiltration (Figure 6A,C,E) and the second diafiltration (Figure 6B,D,F) are represented on the left and right panels, respectively. The second filtration is fed with the concentrated permeate obtained in the first filtration. After the first diafiltrations with the membrane GMT-oNF-2, MELs losses were 36.0% with a purity of 98.3%. However, 66.8% of such MELs were recovered in the second diafiltration (Figure 6 and Table 4).

Remarkably, 88.4% of the MELs fed to the cascade system was collected in the two retentates, and the combined solution presented a purity in MELs of 98.8%. Therefore, the second filtration allowed to reduce cumulative MELs losses to only 11.6%, while attaining high purities, being so far, one of the best results obtained in the literature for MEls purification. Acceptable higher fluxes were obtained, at values of 63.45 L·m^−2^·h^−1^) for EtOAc permeation through GMT-oNF-2, pressurized at 15 bar. EtOAc is an appropriate solvent to extract crude MELs from the fermentation broth. Therefore, this approach is particularly adequate when coupled with our co-substrate feeding strategy A (Table 1) optimized to avoid the presence of TAGs at the end of the fermentation.

The experimental results on MELs purification using the two-diafiltration cascade system and a single diafiltration for PBI 22% are also shown in Figure 4 and Table 4. PBI 22% is a membrane with lower rejections for MELs than GMT-oNF-2. Therefore, the MELs lost to the permeate in the first 4 DV diafiltration were significantly higher for PBI 22%, at values of 58.0% and 61.9% in EtOAc and MeOH than when using the GMT-oNF-2, at a value of 36.0%. Consequentially, in MeOH, the cascade system (using the PBI 22% and 4 DV in each diafiltration) and the single diafiltration approaches (using the PBI 26% with 6 DV) led to similar MELs purities (97.6% vs. 97.1%) and losses (26.3% vs. 32.4%). On the other hand, with EtOAc, the use of 4 DV in two consecutive diafiltrations with OSN 22% yielded a MELs with 93.5% purity, a value below the target threshold. Therefore, implementing the membrane cascade system using the PBI 22% membrane is no benefit.

In resume, this study presents one of the best reported results for MELs purification in EtOAc using the GMT-oNF-2 membrane. This membrane can be used in a single 2 DV diafiltration or in a 4 DV two-diafiltration cascade system to obtain MELs with 90% or 98% purities, respectively, with 15.1% and 11.6% of MELs losses.

For MeOH, the recommended membrane to be used is the PBI 26%, where a MELs purity of 90% or 97% can be reached using 2 or 6 DV in a single diafiltration, with MEL losses of 14.7% or 32.4%, respectively. The use of MeOH is important when TAGs are present in the crude MELs and the selective dissolution step for TAG removal needs to be included on the downstream route. However, the OSN operations are less efficient using MeOH, barely justifying the effort to implement a membrane cascade system, which requires more unit operations than the single membrane diafiltration. These results support the importance of implementing feeding strategies that avoid the presence of TAGs at the end of the fermentation. Moreover, to support the ambition to further purify crude MELs in MeOH, membranes with different features, such as the PBI 24%, could be employed in the cascade system.

### 3.3. Activated Carbon Purification

**Figure 7:**
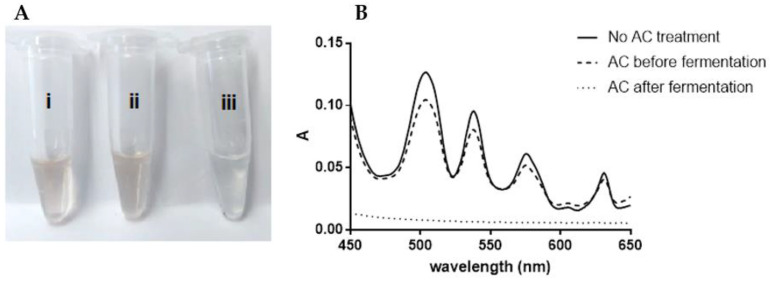
(**A**): Images (**A**) and absorbance spectra (**B**) of solutions of MELs obtained in fermentations using medium prepared with YE with no AC treatment (YE-non treated) or (i) YE treated with AC (YE-AC treated) (ii), and MELs solution treated with AC (MEL-AC treated) (iii), which was obtained from fermentation using medium prepared with YE treated with AC.

Regardless of the purity obtained using the different downstream processing approaches, MELs (also SLs and RMs) have a distinct coloration ranging from yellow to brown. Although for some applications this is acceptable, in other cases, glycolipid decoloration would increase their value. To address this challenge, we considered possible contributions for MELs pigmentation to be associated with: (i) components present in the fermentation medium and/or formed during its sterilization by autoclave; (ii) components or interactions between components generated during the fermentation; and (iii) due to the MEL itself. To gain insight into the source of coloration, the spectra of the visible light wavelength (450–650 nm) range of the medium components and carbon sources (glucose, vegetable oils) solutions were performed before and after sterilization by autoclave. Yeast extract (YE), showed a significant increase in coloration during sterilization, with absorbance increasing at similar wavelengths of the one’s characteristic for crude MELs. Therefore, to further investigate strategies for MEL decoloration, MELs were produced with medium formulated with YE, either as received (YE-non treated) or after to be treated with AC (YE-AC treated). Finally, the MELs, produced using the YE-AC treated, was further dissolved and treated with AC (MELs-AC treated). The MELs obtained were dissolved and the images of the samples and respective spectra obtained are shown in Figure 7.

Interestingly, four distinct peaks are observed in the MELs visible light spectrum. They could probably be attributed to specific compounds that contribute to the coloration of MELs. The total absorption value, within the visible light spectrum, for MELs produced using AC-treated YE was comparable, with a decrease in absorbance of only 8.68%, to the one of produced from YE without any treatment (Table 5). Therefore, it can be concluded that the AC treatment of yeast extract did not significantly reduce the coloration of MELs. On the other hand, when MEL was directly treated by AC, it had virtually complete decoloration, with a decrease in absorbance of 84.88%, when compared to non-treated MEL, even if the MEL used in such experiments was produced with AC-treated YE.

The results of quantification of MEL before and after AC treatment of MELs, shown in Table 6, indicates that MELs and lipids losses are residual during AC treatment.

### 3.4. Comparison of Different Downstream Process for Microbial Glycolipids Purification

The novel downstream process suggested here is compared with other reported approaches for microbial biosurfactant purification in Table 7. Several features of the process are highlighted in such a comparison, namely:

(i) The type of solvent and volumes used, reported in relation to fermentation broth volume harvested;
(ii) The solvent recyclability using a simple distillation was assessed as easy (labeled as “Y”) when no solvent mixtures are formed, the solvent used is of low toxicity and the solvent has an acceptable low boiling point; or difficult (labeled as “N”) when solvent mixtures used formed stable azeotropes, hindering solvent separation by simple distillation, which often ends up as waste streams;
(iii) The number of solvent shifts, i.e., the number of times in the process that the total volume of the solvent is completely evaporated and replaced completely by another solvent, which are high energy-intensive steps; and
(iv) Recovery efficiency, i.e., the weight of product isolated on the end of the downstream route per weight of product produced in the upstream states, in percentage; and
(v) Final purity, i.e., the total weight of the product by the total weight of the product sample.

The method proposed in the present article theoretically enables the complete reuse of solvent used in the downstream process, as mixing multiple solvents is avoided. In the case studies in which the TAGs are not present at the end of the fermentation, crude biosurfactant can be harvested by extraction from the fermentation broth with EtOAc (1:1, thrice) and then purified by OSN also in EtOAc, without any solvent shifts. For such case studies, we recommend the use of a cascade system (4 DV + 4 DV) using a GMT-oNF-2 membrane, followed by AC treatment if color removal is required. The whole process can take place on the same solvent, and if 90% of the solvent used is recycled, a ratio of 1.1:1 of the total volume of EtOAc to fermentation broth will be used.

On the other hand, when residual TAGs are present at the end of the fermentation, TAGs are extracted together with the biosurfactant by EtOAc from the fermentation broth (1:1, thrice). Thus, for such cases, a single solvent shift is necessary from EtOAc to MeOH (0.12:1) and additional MeOH (0.12:1, twice) will be used for removal of TAG by selective dissolution with minimal MELs losses. The three fractions of MELs in MeOH will be added together, with additional methanol to obtain a 50 g/L crude MELs solution that will be fed to OSN. Removal of small lipidic contaminants will be performed by a single diafiltration using a 6 DV using a PBI 26%, followed by AC treatment if color removal is required. This process uses two solvents, EtOAc for the initial harvesting extraction and MeOH for the other unit operations, but do not yield solvent mixtures. When 90% of the solvent used is recycled, a ratio of 1:1 of total solvent volume to fermentation broth will be used. Furthermore, in the suggested process, the use of non-sustainable and toxic solvents is fully avoided.

**Table 7:** The efficiency of downstream methods (LLE—liquid–liquid extraction; CR—crystallization, CP—column purification), indicating the solvent used, the amount of solvent required (volume of solvent/volume of fermentation broth), easiness to recycle solvent used (Y—for easy, N—for difficult), number of solvent shifts, percentage of recovery and final purity (%). A “/” is used to separate information from different unit operations within the same process. “NA” indicates omitted information in the source. At grey are the results obtained here in this study.

Glycolipid	Method	Solvent Used	Solvent Required (Solvent Volume/Broth Volume)	Solvent Recyclability Easiness	No. of Solvent Shifts	Recovery (% *w/w*)	Final Purity (% *w/w*)	Comments	Ref.
RLs	LLE	EtOAc	4:1	Y	1	65.5	84.1	Acid precipitation using HCl, followed by Ethyl acetate LLE	[47]
	LLE + CP	EtOAc	2:1/NA *	N	3	89.4	90.7	LLE with n-hexane, followed by extraction with ethyl acetate. * Solvents used in various ratios for column purification	[48]
		n-hexane	3:1/NA *	N					
SLs	LLE + CP	EtOAc	0.8:1/NA *	N	2	NA **	NA **	* Column chromatography used for purification, solvent consumption not reported ** Recovery rate and final purity not reported	[49]
		2-propanol	0.2:1	N					
		MeOH	NA *	N					
	LLE + CP	EtOAc	0.8:1/NA *	N	3	NA **	NA **	Oleic acid used as lipophylic substrate LLE with Ethyl acetate/2-propanol mixture, extract washing with hexane, followed by column chromatography * Solvent consumed for column purification not reported ** Recovery rate and final purity not reported	[50]
		2-propanol	0.2:1	N					
		n-hexane	1:1	Y					
		Chloroform	NA *	N					
		MeOH	NA *	N					
MELs	LLE	MTBE	3:1	Y	3	8	100	Multiple solvents used in subsequent extractions	[25]
		MeOH	1.6:1	N					
		Cyclohexane	3:1	N					
		n-hexane	1:1	N					
	LLE + CP Preparative HPLC	EtOAc	2:1	Y	3	4 **	100	* Two column separations and preparative HPLC using CHCl_4_/MeOH ratios 90/10, 95/5, 96/4. Unknown volumes, non-recyclable ** Reported recovery based on mass of MELs after L–L extraction	[22]
		MeOH	1:1/ NA *	Y; N *					
		Chloroform	NA *	N *					
	LLE + CP	EtOAc	1:1	Y	2	50 **	100	* Column purification with gradient elution by CHCl_4_/Acetone mixture (80:20, 40:60 and 0:100, *v/v*). Volume unknown, most likely immense, non-recyclable ** Reported recovery based on mass of MELs after L-L extraction	[23]
		Chloroform	NA *	N					
		Acetone	NA *	N					
	LLE	MeOH	2.5:1	N	1	90	100	Multiple solvents used in subsequent extractions; solvent shifts avoided	[26]
		n-hexane	3:1	N					
	LLE + OSN + AC treatment	EtOAc	21	Y	0	88.4	98.8	Method reported in this article. No TAG present. EtOAc used through all the process. Cascade system (4 DV + 4 DV), GMT-oNF-2 membrane. 90% of solvent recycled	
	LLE + TAG removal + OSN + AC treatment	EtOAc + MeOH	1:1	*Y*	1	64.8	97.1	Method reported in this article. TAG present. EtOAc used in extraction. MeOH used through the other steps of the process. Single diafiltration (6 DV), PBI 26% membrane. 90% of solvent recycled	

## 4. Conclusions

For the first time, this study presents a unique method, based on OSN, for the purification of glycolipid biosurfactants that uses only one solvent and reaches a 98% purity, with product losses of about 11.6% and the possibility of solvent recycling.

Different commercially available and homemade membranes (made of polybenzimidazole (PBI) were selected to retain MELs, while allowing permeation of smaller lipidic contaminants (FFA and MAG). None of the assessed OSN membranes presented an interesting performance for the targeted separation. While, some have low rejections for MELs (67.7 to 87.1%), the ones with high retentions to MELs (rejections above 97%) also present high rejections for the lipids (above 70%), such as the case of the PBI 26% membrane. According to the model presented in our previous study [45], such membranes can be used to obtain a high product purity, but at costs of high product losses (20–40%) and use of high DV.

Different case studies are evaluated. A first case study highlights the importance of optimizing fermentations to avoid the presence to triacylglycerols (TAGs) at the end of the fermentation. In such cases, only one solvent, EtOAc, was used for the whole process. The PBI 26% can effectively be used in diafiltrations with 2 DV or 6 DV, respectively, to reach purities of 90% or 97% with MELs losses of 15% or 58%. While such a strategy is interesting to reach the lower purity grade, the MELs losses that require attaining 97% purity are prohibitive. Therefore, a sequential two-filtration diafiltration, each with 4 DV, was implemented using a GMT-oNF-2. Such a membrane has lower rejections for the small lipids, so MELs purifications will be facilitated. However, its rejection of MELs is also quite low and, thus, the permeate of the first diafiltration is fed to a second OSN is to recover the MELs lost. Remarkably, for GMT-oNF-2, it is possible to recover 67.8% of MELs lost in the first filtration, achieving an overall MELs purity of 98% with losses of 11.6%. So far, this is one of the best values reported in the literature for downstream of microbial biosurfactants. It is noteworthy that as this downstream route only uses one solvent, its recycling by simple distillation will be facilitated.

Further reduction of solvent volume used can be relatively easy and significantly optimized, against solvent flux against the membrane, by increasing the concentration of crude MELs used in the OSN to values above 50 g/L (e.g., 150 g/L). Moreover, performing only two extractions of the fermentation broth will also reduce solvent use (MELs contents on the third extraction were residual, data not shown). However, the implementation of further solvent volume reduction in the harvesting extraction step is challenging and deserves dedicated research attention.

A second interesting case study considers the presence of TAG on the end of the fermentation. This corresponds to many of the scenarios reported on the literature, as large amounts of vegetable oils are used as carbon sources to attain high glycolipids biosurfactant production. To address such separation requirements, an additional operation unit was successfully developed for the removal of 90% TAGs, with minimal losses of only 4.1% of MELs. In this process route, to avoid further solvent shifts, the process route for removal of other lipid derivatives (FFA, MAG and DAG) by OSN was processed in MeOH. Unfortunately, for diafiltrations in MeOH, the limitations posed by the sub-optimal performance of the membranes could not be circumvented using the two-filtration cascade system. Still, our homemade PBI 26% membrane could be used in MeOH to obtain, in a single diafiltration of 2 DV or of 6 DV, MELs purities of 90% or 97%, with losses of 15% and 32%, respectively. Note that the process described here can be further improved using membranes with rejections slightly higher MEL or lower for residual MELs, respectively, in the single diafiltration or cascade system modes.

A final purification step with activated carbon for the removal of color was implemented. While, in the first instance, yeast extract drew our attention, we could not assert its responsibility. Instead, this study shows that other contaminants originated during fermentation could clearly be removed from the crude MELs samples without losses of this product.

Overall, the proposed downstream process for glycolipid biosurfactants was tailored to reduce solvent waste streams and avoid or mitigate solvent shifts. This resulted in a downstream process that is potentially more sustainable, as solvent streams based on a single solvent facilitates their recyclability by distillation.

## Figures and Tables

**Table 1:** Composition of crude MELs obtained in the end of fermentation, based on the feed strategy employed.; Glu—glucose; SBO—soybean oil; FFA—free fatty acids; MAG—monoacylglycerides; DAG—diacylglycerides; TAG—triacylglycerols.

Feed Strategy	Time (Days)	MELs (%)	FFA (%)	MAG (%)	DAG (%)	TAG (%)
(A) 40 g/L Glu (day 0) + 20 g/L SBO (day 0, 4)	9	84.6	6.9	8.3	-	-
(B) 40 g/L Glu (day 0) + 20 g/L SBO (day 4, 7)	12	62.0	11.0	23.0	2.0	-
(C) 80 g/L SBO (day 0)	6	56.3	3.3	6.4	0.2	33.8

**Table 2:** Rf values of MELs and SBO for different organic solvents. Solvents with ΔRf higher than 0.50 and higher than 0.75 are highlighted in green and dark green, respectively, indicating potential higher and superior separation between MELs and SBO.

Eluent	Rf SBO	Rf MELs	ΔRf
IPA	0.79	0.58	0.21
Chloroform	0.80	0.00	0.80
MTBE	1.00	0.39	0.61
MeOH	1.00	0.60	0.60
EtOAc	0.94	0.67	0.27
DCM	0.89	0.00	0.89
Hexane	0.00	0.00	0.00
Water	0.00	0.00	0.00
EtOH	0.00	0.73	0.73
Acetone	0.85	0.56	0.29

**Table 3:** Cumulative MELs and contaminant losses and composition of combined top phases for the consecutive step of MEL purification with MeOH by solvent-selective dissolution).

		Initial Sample	After 1st Step	After 2nd Step	After 3rd Step
	MELs Losses (%)	-	12.3	5.9	4.1
	TAG removed (%)	-	94.7	91.2	90.6
Composition	MELs (%)	39.0	66.0	63.9	62.8
	TAG (%)	39.0	4.0	6.0	6.1
	FFA + MAG + DAG (%)	22.0	30.0	30.1	31.0

**Table 4:** MELs losses and purity (%) for different OSN membranes (GMT-oNF-2, PBI 22 and 26%) and both filtrations when using EtOAc or MeOH as organic solvents. In the cascade system, two diafiltrations with 4 DV in each (membranes GMT-oNF-2 and PBI 22%) were used, and on the single diafiltration 6 DV (PBI 26%) was used.

Type of Filtration	Organic Solvent	EtOAc			MeOH	
	OSN	GMT-oNF-2	PBI 22%	PBI 26%	PBI 22%	PBI 26%
	Diavolumes Used	4 + 4	4 + 4	6	4 + 4	6
1st filtration	MELs losses (%)	36.0	58.0	26.3	61.9	32.4
	MELs purity (%)	98.8	93.7	98.0	97.5	97.1
2nd filtration	MELs losses (%)	32.2	27.8	-	47.2	-
	MELs purity (%)	98.6	93.2	-	97.7	-
	MELs recovered (%)	67.8	72.2	-	52.8	-
Overall	MELs losses (%)	11.6	16.1	26.3	29.2	32.4
	MELs purity (%)	98.8	93.5	98.0	97.6	97.1

**Table 5:** Absorbance reduction due to AC treatment of YE or AC of the crude.

	MELs from YE-AC treated per MELs from YE-non treated	MELs*-*AC treated per MELs from YE-AC treated
Percentage ratio of absorbances (%)	91.32	15.12%

**Table 6:** Analysis of crude MELs composition before and after treatment with AC. MELs used were produced using AC-treated YE.

Compounds (g/L)	MELs before AC Treatment	MELs after AC Treatment
MELs	49.90 ± 2.13	49.12 ± 2.04
Residual lipids	0.61 ± 0.28	0.70 ± 0.45

## Data Availability

Data available on request.

## Supplementary Materials

The following are available online at: https://www.mdpi.com/article/10.3390/membranes13010081/s1. Figure S1: TLCs with different solvents. Left dot—soybean oil. Right dot—MELs (with some residual fatty acids). A—isopropanol; B—chloroform; C—MTBE; D—methanol; E—ethyl Acetate; F—DCM; G—hexane; H—water; I—EtOH; J—acetone; Figure S2: TLC with methanol as eluent. FFA—partially hydrolysed oil with free fatty acids; Figure S3: Results for three-step separation with methanol; Figure S4: Ratio of permeate/feed of different fatty acids (C8, C10, C12, C14, C16, C18) chains for different membranes, using MeOH (A) and EtOAc (B) as organic solvents; Figure S5: HPLC chromatogram of SL sample diluted in methanol. Black line—initial sample. Purple line—retentate sample after 2 DV. Cyan line—retentate sample after 6DV. Table S1: Experimental values of MEL, residual lipids rejection (%) and flux (L/m^2^/h/bar) for each membrane tested using EtOAc or MeOH as organic solvents. For each membrane, we also calculated the theoretical minimum DV and the corresponding MEL losses (%) to achieve 97% of purity.
