# Supplementary material for: Novel Organic Solvent Nanofiltration Approaches for Microbial Biosurfactants Downstream Processing
Source: Membranes (Basel). 2023 Jan 9;13(1):81. doi: 10.3390/membranes13010081 (PMC9860925; doi:10.3390/membranes13010081)
Supplement: Supplementary file 1 [file membranes-13-00081-s001.zip › membranes-2012809-supplementary.pdf]

membranes

Supplementary Information

Novel Organic Solvent Nanofiltration Approaches for Microbial Biosurfactants Downstream Processing

Miguel Figueiredo Nascimento [1,2], Petar Keković [1,2,3], Isabel A. C. Ribeiro [4], Nuno Torres Faria [1,2] and Frederico Castelo Ferreira [1,2,*]

[1] Department of Bioengineering, iBB—Institute for Bioengineering and Biosciences, Instituto Superior Técnico, Universidade de Lisboa, Av. Rovisco Pais, 1049-001 Lisboa, Portugal
[2] Associate Laboratory, i4HB—Institute for Health and Bioeconomy, Instituto Superior Técnico, Universidade de Lisboa, Av. Rovisco Pais, 1049-001 Lisboa, Portugal
[3] MIT-Portugal Program, 77 Massachusetts Avenue, Cambridge, MA 02139, USA
[4] Research Institute for Medicines (iMed.ULisboa), Faculty of Pharmacy, Universidade de Lisboa, Av. Prof. Gama Pinto, 1649-003 Lisboa, Portugal
* Correspondence: frederico.ferreira@tecnico.ulisboa.pt; Tel.: +351-218419598

Supplementary information

Solvent interaction with MELs and lipids

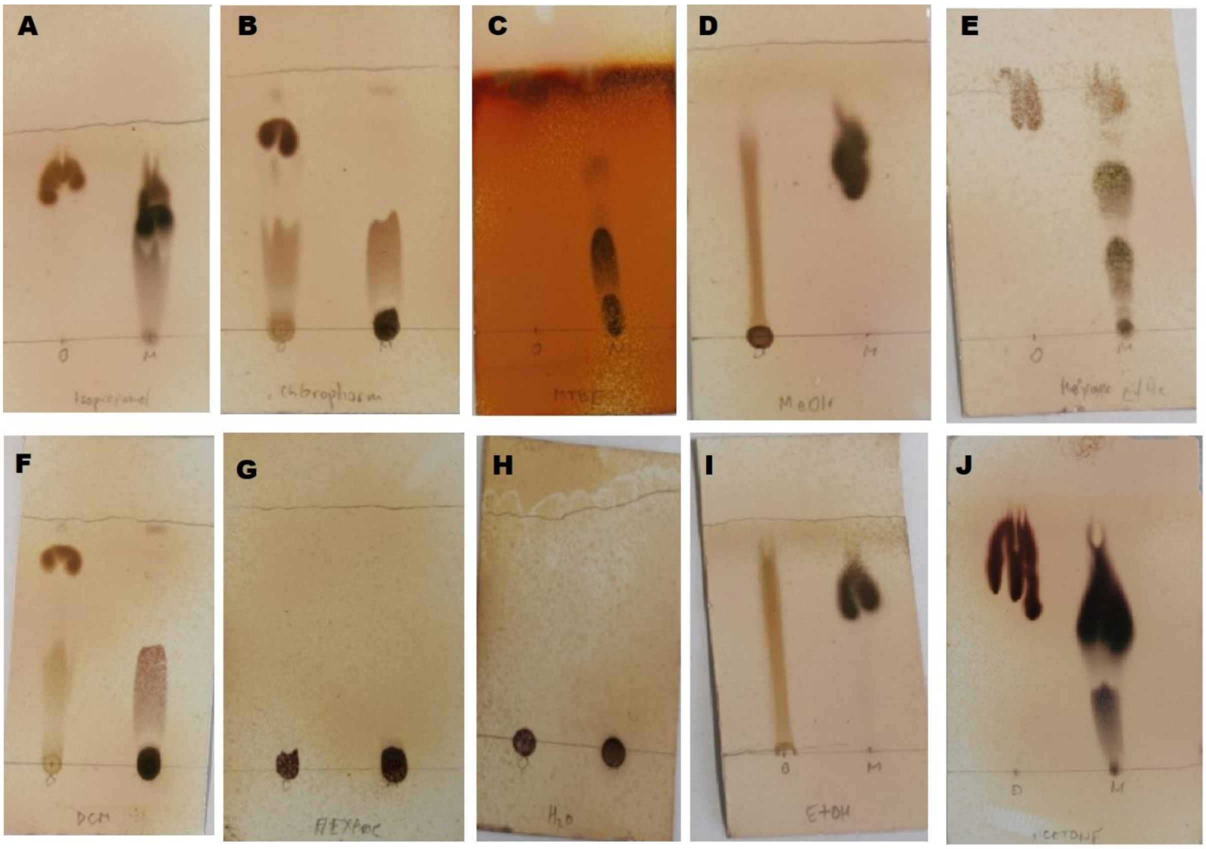

Figure S1: TLCs with different solvents. Left dot - Soybean oil. Right dot - MELs (with some residual fatty acids). A - Isopropanol; B - Chloroform; C - MTBE; D - Methanol; E - Ethyl Acetate; F - DCM; G - Hexane; H - Water; I - EtOH; J – Acetone

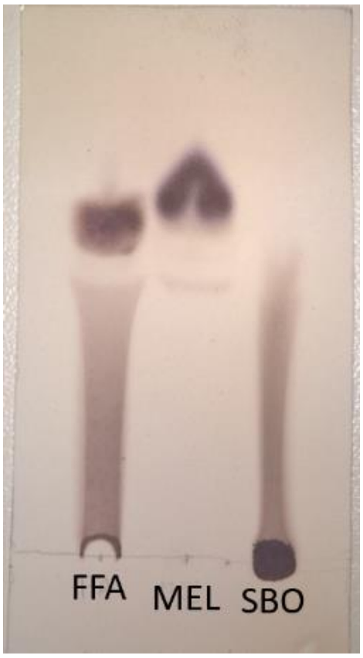

Figure S2: TLC with methanol as eluent. FFA - partially hydrolysed oil with free fatty acids

The role of Methanol

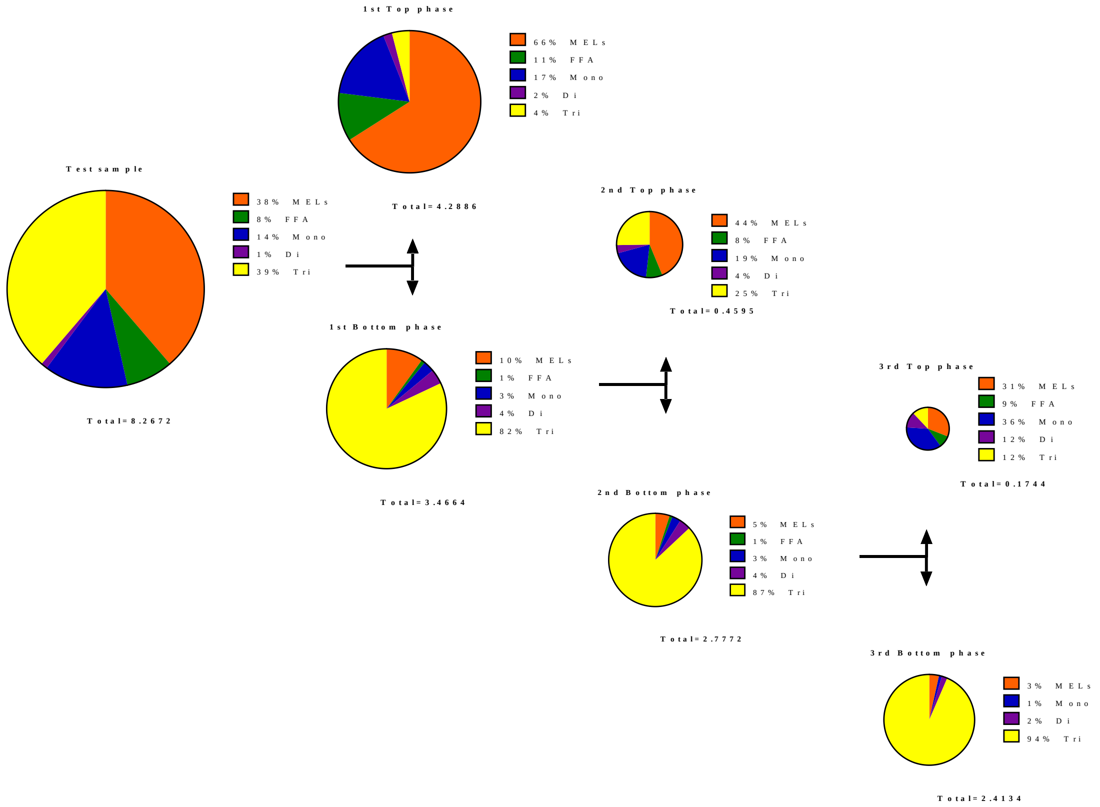

Figure S3: Results for three-step separation with methanol

OSN screening and diavolumes

Table S1: Experimental values of MELs, residual lipids rejection (%) and flux (L/m²/h) for each membrane tested using EtOAc or MeOH as organic solvents. For each membrane it was also calculated the theoretical minimum DV and the correspond MEL losses (%) to achieve 97% of purity. The membranes marked at [1] means that to the slow flux, only 10 mL were permeated, instead of 25, and 30 bar of pressure was used.

Solvent	Membrane	MELs Rejection (%)	Residual lipids rejection (%)	Flux (L/m²/h)	Theoretical Minimum DV (-)	Theoretical MELs losses (%)
EtoAC	GMT-oNF-2	87.1 ± 0.6	32.0 ± 0.4	69.0	3	32.0
	PuraMem- 600	84.3 ± 4.5	38.4 ± 21.5	25.5	3	38.1
	PBI 22%	73.1 ± 0.5	32.6 ± 8.4	36 ± 1.5	4	66.00
	PBI 22%-X	78.4 ± 0.5	60.8 ± 3.6	33 ± 1.5	7	78.5
	PBI 24%	92.3 ± 4.0	68.0 ± 7.3	16.5 ± 0.0	7	38.1
	PBI 24%-X	84.0	56.0	21 ± 0.0	6	61.7
	PBI 26%	97.0 ± 1.0	71.0 ± 3.0	9.0	6	16.5
	PBI 26%-X[1]	99.2 [1]	96.3 [1]	2.7	-	-
MeOH	DuraMem-500	88.4 ± 0.7	74.9 ± 6.2	27 ± 0.0	9	66.0
	PBI 22%	67.7 ± 9.5	26.6 ± 12.3	39.5 ± 1.5	4	73.3
	PBI 22%-X	83.1 ± 0.9	60.1 ± 5.2	37.5 ± 0.0	5	69.5
	PBI 24%	93.0 ± 2.0	52 ± 10.2	43.5 ± 1.5	5	24.2
	PBI 24%-X	93.3 ± 1.3	67.8 ± 1.7	34.5 ± 3.0	7	18.9
	PBI 26%	97.1 ± 0.9	75.1 ± 8.7	30 ± 3.0	7	18.9
	PBI 26%-X	98.1 ± 0.8	77.5 ± 3.6	25.5 ± 3.0	8	14.7

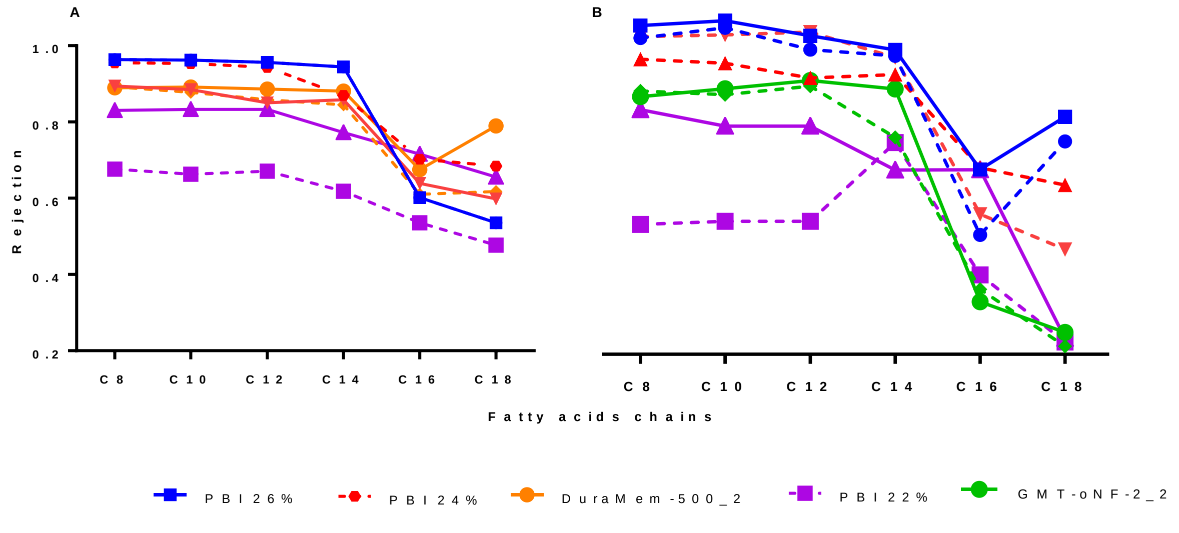

Figure S4: Rejection of different OSN membranes for MELs (C8, C10, C12 and C14) and residual lipids (C16 and C18), using MeOH (A) and EtOAc (B) as organic solvents. The existing species on permeate and feed are submitted to meth-anolysis and the obtained concentrations of methyl esters obtained in permeate (C_P) and feed (C_F) and rejections are calculated as 1- C_P/C_F.

SL nanofiltration

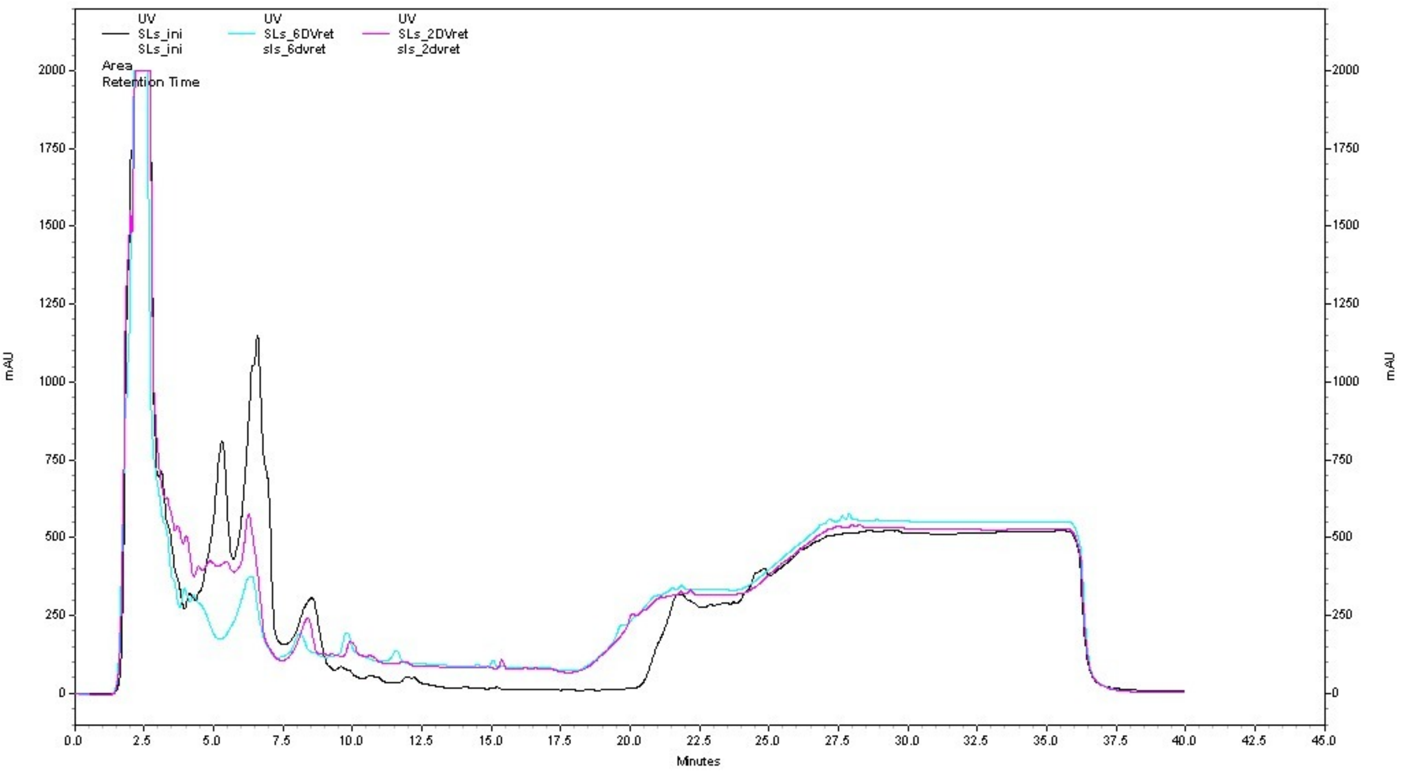

Figure S5: HPLC chromatogram of SL sample diluted in methanol. Black line – initial sample. Purple line – retentate sample after 2DV. Cyan line – retentate sample after 6DV.

The used HPLC method is able to discriminate between individual lipid groups, with free FFA occurring between t = 2.5-7.5 min, MAG between t = 5-20 min, DAG between t = 20-25 min and TAG between t = 25-35 min. One can be observed a reduction of the peaks on the region of the FFA and MAG. From the chromatogram it can be observed that the nanofiltration of SLs successfully removed small lipidic contaminants from the crude SL mixture.
